# Supplementary material for: CRISPR-enhanced human adipocyte browning as cell therapy for metabolic disease
Source: Nat Commun. 2021 Nov 26;12:6931. doi: 10.1038/s41467-021-27190-y (PMC8626495; doi:10.1038/s41467-021-27190-y)
Supplement: Supplementary file 1 — Supplementary Information [file 41467_2021_27190_MOESM1_ESM.pdf]

Supplementary Information

CRISPR-enhanced human adipocyte "browning" as cell therapy for metabolic disease

Emmanouela Tsagkaraki[1,2]*, Sarah M. Nicoloro[1]*, Tiffany DeSouza[1], Javier Solivan-Rivera[1], Anand Desai[1], Lawrence M. Lifshitz[1], Yuefei Shen[1], Mark Kelly[1], Adilson Guilherme[1], Felipe Henriques[1], Nadia Amrani[2], Raed Ibraheim[3], Tomas C. Rodriguez[3], Kevin Luk[4], Stacy Maitland[4], Randall H. Friedline[1], Lauren Tauer[1], Xiaodi Hu[1], Jason K. Kim[1,5], Scot A. Wolfe[4,6], Erik J. Sontheimer[1,3,6], Silvia Corvera[1]**, Michael P. Czech[1]**.

[1]Program in Molecular Medicine, University of Massachusetts Medical School, Worcester, MA 01605, USA
[2]University of Crete School of Medicine, Crete 71003, Greece
[3]RNA Therapeutics Institute, University of Massachusetts Medical School, Worcester MA 01605, USA
[4]Department of Molecular, Cell and Cancer Biology, University of Massachusetts Medical School, Worcester MA 01605, USA
[5]Division of Endocrinology, Metabolism and Diabetes, Department of Medicine, University of Massachusetts Medical School, Worcester, MA 01605, USA
[6]Li Weibo Institute for Rare Diseases Research, University of Massachusetts Medical School, Worcester, MA 01605, USA

*These authors contributed equally
**Co-corresponding authors

Michael P. Czech (Michael.Czech@umassmed.edu)
Silvia Corvera (Silvia.Corvera@umassmed.edu)

Contents

Supplementary Figure 1

Supplementary Figure 2

Supplementary Figure 3

Supplementary Figure 4

Supplementary Figure 5

Supplementary Table 1

Supplementary Table 2

Supplementary Figure 1

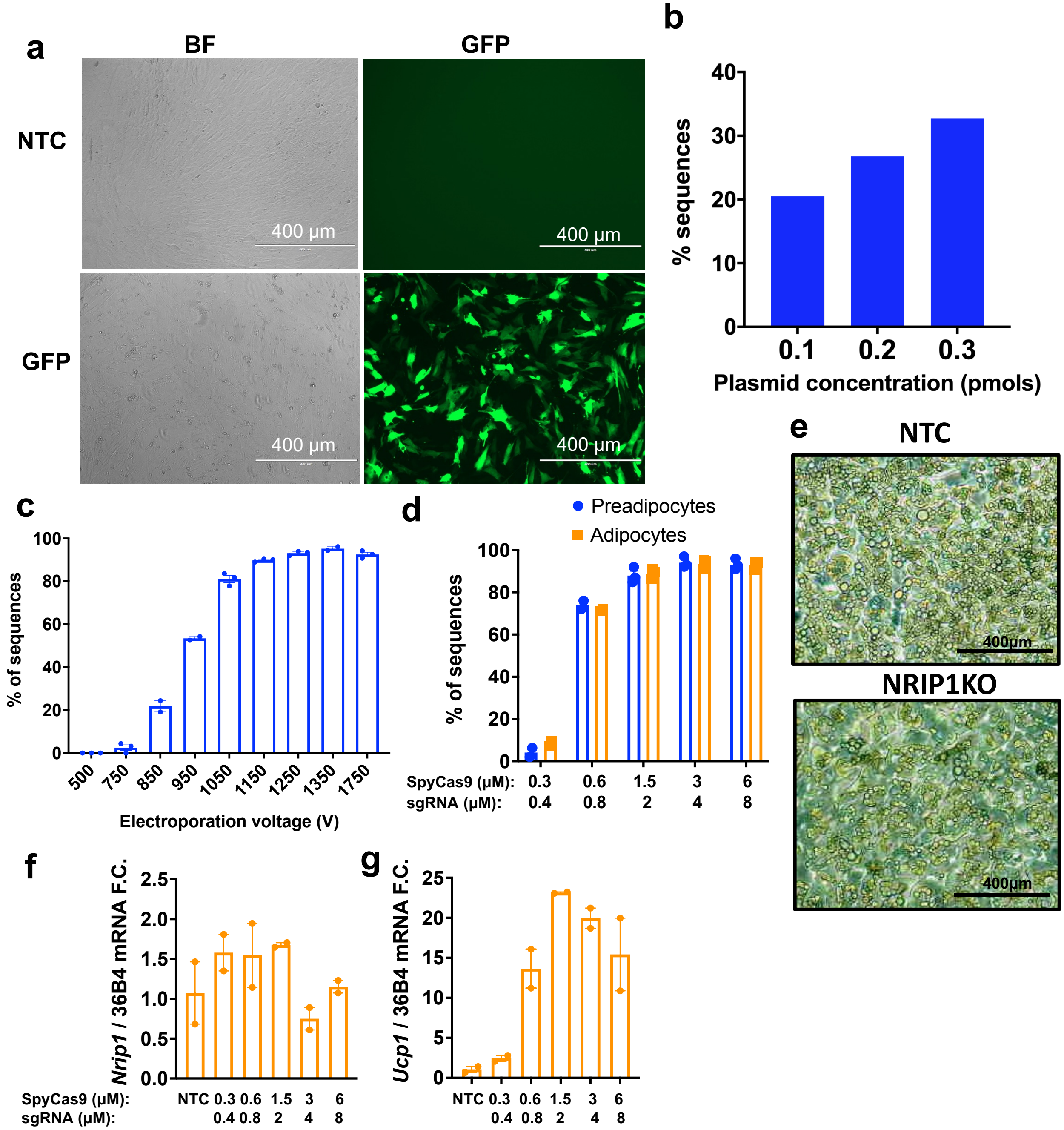

Supplementary Fig. 1. SpyCas9/sgRNA RNPs are more efficiently delivered into murine adipocytes by electroporation than plasmid expression. Delivery of SpyCas9/sgRNA RNPs versus plasmids encoding either GFP or Cas9 and sgRNA electroporated with preadipocytes were compared. **a**. GFP expression plasmid (Lonza pmaxGFP) was used in various concentrations for optimization of the plasmid delivery by

electroporation in murine preadipocytes to determine the optimum range (0.1-0.2 pmols) that induces GFP expression by fluorescent microscopy (white line = 400 μm). Top: Control preadipocytes Bottom: Preadipocytes transfected with 0.2 pmols at 72 hours after electroporation (1350 V, 30 ms, 1 pulse). **b.** Application of the optimized electroporation protocol to transfect preadipocytes with plasmids expressing SpyCas9 and sgRNA-M6 in three different concentrations within the range determined with the GFP plasmid titration: 0.1 - 0.3 pmols. **c.** Optimization of the electroporation protocol to deliver CRISPR RNPs in preadipocytes with 1 pulse and 30 ms width and increasing voltage**. d.** Titration of RNP concentrations in correlation with the editing efficiency with sgRNA-M6. **e.** Mature adipocytes transfected before differentiation with either NTC RNPs or sgRNA-M6 in cell culture and magnification 10X. **f.** *Nrip1* gene expression by RT-PCR in mature adipocytes transfected with varying RNP concentrations. **g.** *Ucp1* gene expression by RT-PCR in mature adipocytes transfected with varying RNP concentrations.

NTC = Non-targeting control. Bars denote mean. Error bars denote Mean ± S.E.M. n ≥ 3 biologically independent replicates. Detailed n per condition is shown in source data file. *Nrip1* sgRNA-M4 was used in the titration experiment.

Supplementary Figure 2

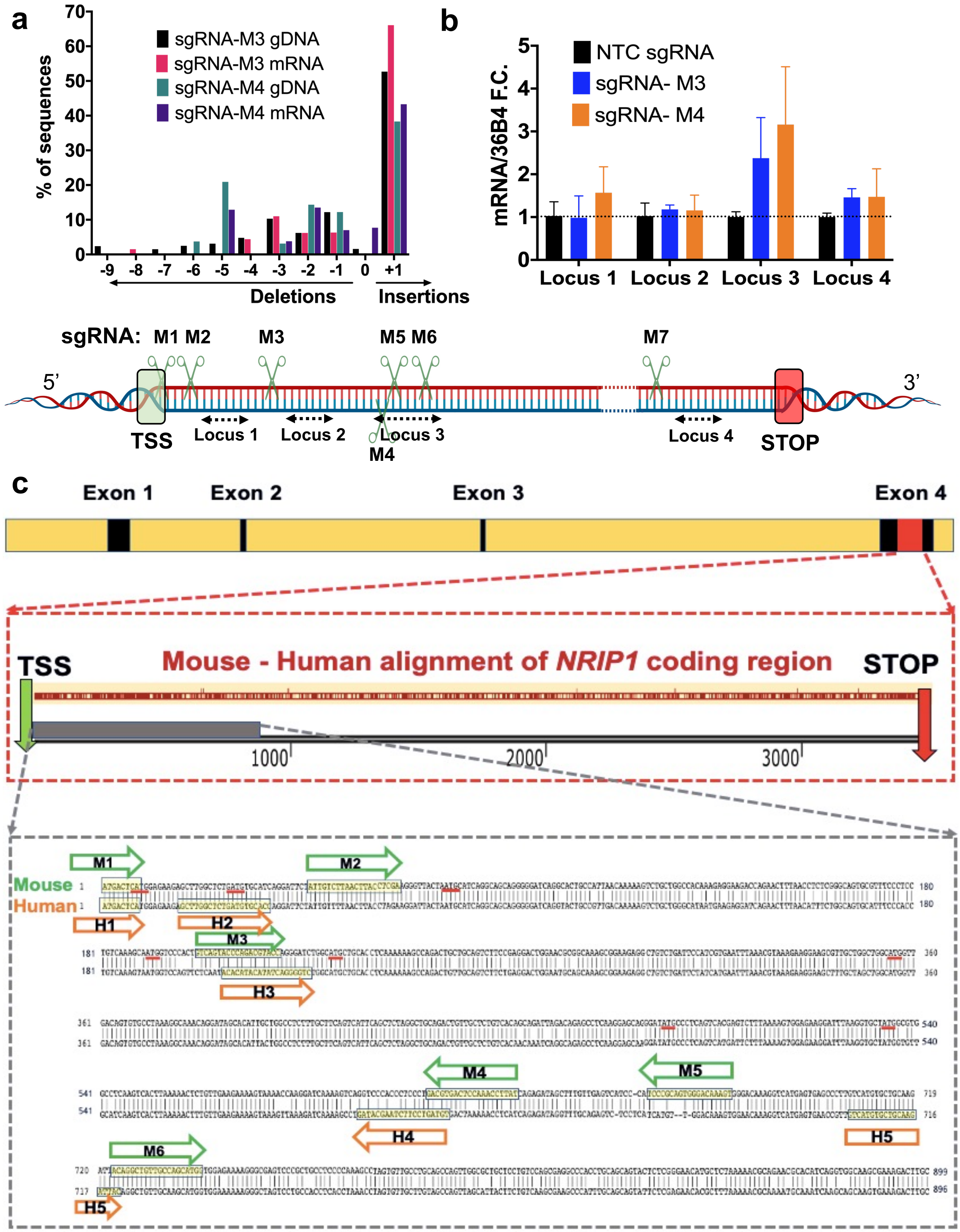

Supplementary Fig. 2. mRNA of *Nrip1* harbors the indels created by the CRISPR-RNPs with little evidence of truncation or degradation of modified *Nrip1* mRNAs. **a.** Comparison of the indel distribution of the genomic DNA and the cDNA after double rDNase treatment in the template RNA of cells transfected with sgRNA-M3 and sgRNA-M4. **b.** RT-PCR results of the expression of different loci across *Nrip1* cDNA as shown in the map. **c.** Top: *Nrip1* gene includes 4 exons (black) and the coding region (red) is contained in exon 4. Middle: Alignment of the mouse and human coding regions of *NRIP1* that spans 3486bp and 3477bp respectively between the TSS and STOP codons, highlighting the site targeted with sgRNAs M1-6 and H1-5 (gray). Bottom: 5'→ 3' DNA sequence alignment of the mouse (top strand) and human (bottom strand) of the N-terminus area targeted with the sgRNAs M1-6 for the mouse (green arrows) and H1-5 for the human (orange arrows). Underlined (red) are the ATG sequences in frame or out of frame between the TSS and the first sgRNA that depletes NRIP1 which could potentially serve as alternative translation initiation sites. In b error bars denote mean ± S.E.M. n ≥ 3 biologically independent replicates. Precise n per condition is shown in source data files.

Supplementary Figure 3

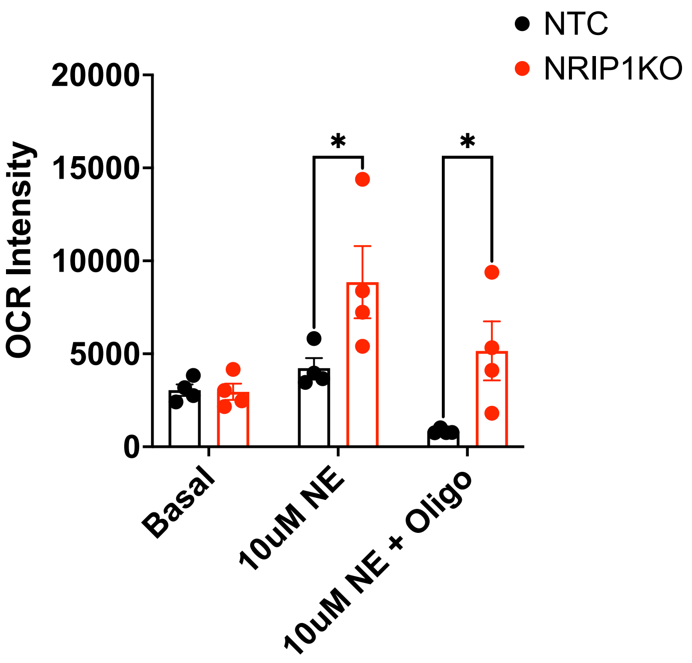

Supplementary Fig. 3. Biological replicates showing primary mouse NRIP1KO adipocytes display higher rates of uncoupled oxygen consumption than NTC adipocytes. Oxygen consumption rates (OCR) in mature cultured primary adipocytes targeted with NTC or NRIP1 sgRNA-M6 without norepinephrine (NE) stimulation (Basal) or with NE stimulation or with norepinephrine plus oligomycin (NE oligo). Statistical comparison for OCR was performed using two-way ANOVA with Sidak's multiple comparison test. Values presented represent 4 biological replicates, each performed with triple technical replicates combining both male and female primary adipocytes. NE $p = 0.0203$, NE oligo $p = 0.030$. Bars denote mean and error bars denote mean ± S.E.M.

Supplementary Figure 4

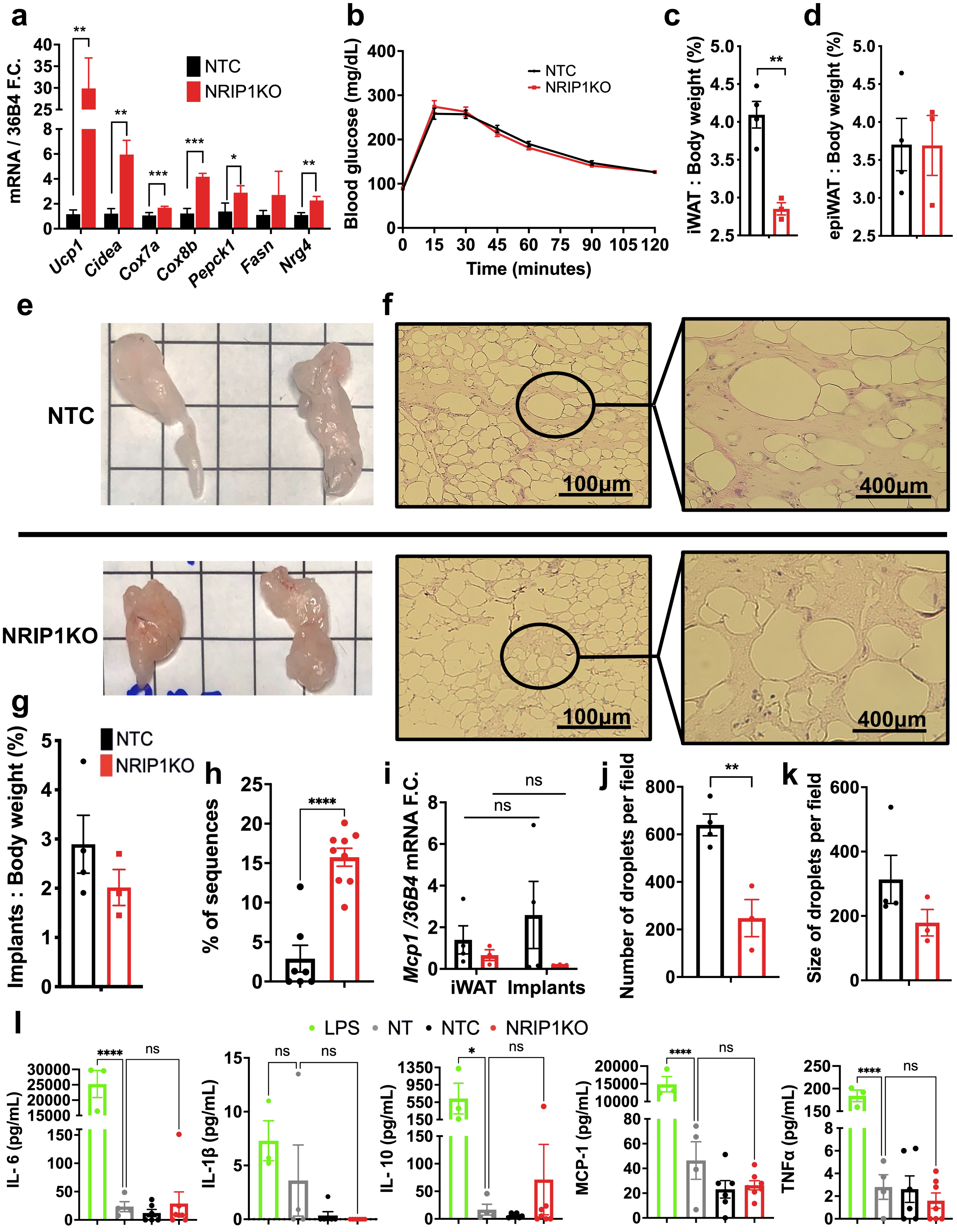

Supplementary Fig. 4. Characterization of mice implanted with Cas9/NTC sgRNA-

versus Cas9/sgRNA-M6-treated adipocytes. a. RT-PCR results for expression of genes involved in thermogenesis, mitochondrial electron transport chain, lipid and glucose metabolism and the neurotrophic factor Nrg4 prior to implantation. P values: *******Ucp1* = 0.004; *******Cidea* = 0.003; ****Cox7a* = 0.0018; ****Cox8b* = 0.000005; **Pepck1* = 0.037; ***Nrg4* = 0.006. **b.** Baseline GTT after 16-hour fasting in the chow-fed recipients before the implantation of adipocytes. **c**. iWAT over whole body weight percentage. P value = 0.002. **d**. epiWAT over whole body weight percentage. P value = 0.983. **e.** Macroscopic images of the whole implants of the recipients after dissection (square = 1cm^2). **f.** Histology images stained with H&E of the implant in 5X magnification (left) and 20X magnification (right). **g.** Implants over whole body weight percentage. **h.** Editing evaluation of homogenized implant sample for each of the NRIP1KO adipocyte recipients by Sanger sequencing. P value = 0.00001. **i.** MCP-1 mRNA expression by RT-PCR in implants after dissection. **j.** Quantification of the number (P value = 0.006) and **k.** size of lipid droplets (P value = 0.216) in the histology of the livers of the mouse NRIP1KO adipocytes recipients. **l.** Measurement of cytokines/chemokines in the plasma of NTC and NRIP1KO implant recipients, non-treated controls on HFD and LPS injected mice (1μg per mouse) for 2 hours. P values IL-1β = 0.014; IL-6 < 0.0001; IL-10 = 0.0095; MCP1 < 0.0001 by one – way ANOVA.

Black = NTC cell implant recipients; red = NRIP1KO cell implant recipients, in a-b: NTC (n=13) ; NRIP1KO (n=14), g, j, k NTC (n=4) ; NRIP1KO (n=3) ; LPS (n=3) ; NT (n=4); NTC (n=6), NRIP1KO (n=7). Each n represents number of biologically independent mice. Bars denote mean, error bars denote mean ± SEM. *p < 0.05, **p < 0.01 by unpaired two-tailed T-test unless otherwise specified.

Supplementary Figure 5

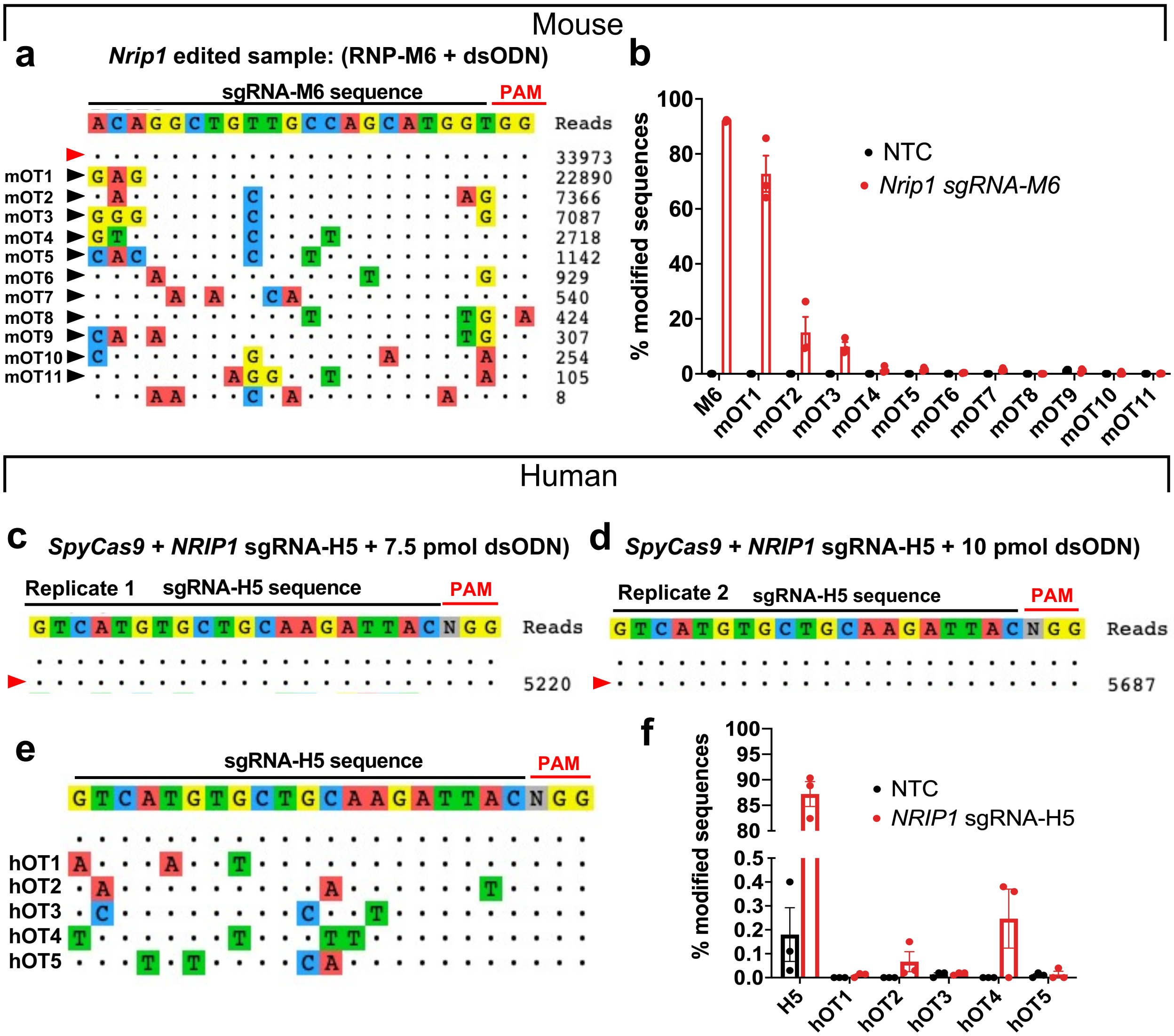

Supplementary Fig. 5. Off-target screen by GUIDE-seq and amplicon NGS sequencing in mouse and human adipocytes. a. Mouse predicted off-target sites detected by GUIDE-seq in NRIP1KO mouse preadipocyte library (sgRNA-M6 + SpyCas9 + 7.5 pmol of dsODN) with up to 6 sgRNA-M6 mismatches and up to 1 PAM mismatch ranked by number of reads. Red triangle points the on-target site, and black triangles show the 11 top off-target sites ranked by number of mismatches and selected for Amplicon NGS screening. **b.** Indel quantification by amplicon next generation sequencing on mouse mature adipocytes on-target (M6) and selected off-target loci (mOT1-11). **c.** Human predicted sites with DSB detected by GUIDE-seq in NRIP1KO human progenitor library (sgRNA-H5 + SpyCas9 + 7.5 pmol of dsODN) with up to 6

sgRNA-H5 mismatches and up to 1 PAM mismatch ranked by number of reads. Red triangle points the on-target site. **d.** Human predicted sites with DSB detected by GUIDE-seq in NRIP1KO human progenitor library (sgRNA-H5 + SpyCas9 + 10 pmol of dsODN) with up to 6 sgRNA-H5 mismatches and allowed NGG, NAG, NGA PAM sequences. Red triangle points the on-target site. **e.** Selected candidate off-target sites of sgRNA-H5 on human genome by Cas-offinder software. **f.** Indel quantification by amplicon next generation sequencing on human mature adipocytes on-target (H5) and selected off-target loci (hOT1-5). In amplicon NGS, NTC (n=3), NRIP1KO (n=3) where n represents biologically independent replicates. Error bars denote mean ± S.E.M.

Supplementary Table 1. Primers for Sanger sequencing (sequencing primers bolded) and Amplicon NGS PCR1.

**Mouse Sanger Sequencing primers (5'→ 3')**	
M1-3 F_1	**CTCATCAGTGCTGTAGTCTG**
M1-3 F_2	**TGTGCTAAGGAAGTTGCGAGG**
M1-3 R	AGCCTAGAGCTGAATGACT
M4-6 F	**CTCTAGGCTGCAGACTGTT***
M4-6 R	**CAGTGAGTTCTTATAGCTCG****
M7 F	**CTTCTTAGGTGTGACTCCC**
M7 R	CTATGATCCGTCACACAAC
**Mouse Amplicon NGS primers (5'→ 3')**	
M6 F<br>M6 R	AAAAGTCAGGTCCCACCCT<br>GTGCGTTCTGCGTTTTTAGA
mOT1 F<br>mOT1 R	CGTCCTATACGCTTGGTGTTG<br>TGCGTATGAGTGTTTTGCTTGC
mOT2 F<br>mOT2 R	TGGGTAACTAGAGAGATGGTTCAG<br>TCAGTAGGCCTCATGGAAA
mOT3 F<br>mOT3 R	GTTGGCCATTACCCATCTTTCA<br>ATAAAATACAAGTCCCGGGCTGCTG
mOT4 F<br>mOT4 R	TTGAAATCTGGAGTGACTGG<br>GATAAGCCTCAAAATTACCCCC
mOT5 F<br>mOT5 R	CACCGGCCAGATTACATTTT<br>GGTAACTGAATTGGAACGCA
mOT6 F<br>mOT6 R	TGCAGACATGTTCATCCCTC<br>CATCACCATGGGAACCAGA
mOT7 F<br>mOT7 R	CCCTACAACTTTGTGACTTTCC<br>TGTGACCAACAAAGTGTCCC
mOT8 F<br>mOT8 R	AGTGTCATCAAGCTGCAGGAG<br>TCCAATCCGGTACAAACCAT
mOT9 F<br>mOT9 R	GTTTTTCCCAATCCCATTCC<br>TCCAGAGACTGAACCACCAAC
mOT10 F<br>mOT10 R	ACAGAACAGATGGCAGACGG<br>CAACTTGATGGGAGTTTCAGC
mOT11 F<br>mOT11 R	TGTCGGACGTACGTTGTTCT<br>ACAGGACACACTTAGCAGCGT

**Human Sanger Sequencing primers (5'→ 3')**	
H1-H2 F	**CTTCTCCTCCTCCTTGCGTA**
H1-H2 R	CTTGAGGCTCTGCCTGATTT
H3 F	**AGTTACATCGCACTCACC**
H3 R	GGAGACTCTGCAAACCTAT
H4 F	CACCTGTCAAAGTAATGGTC
H4 R	**CACTAGGTTTAGGTGAGGTG**
H5 F	**TAGCACATTACTGGCCTC**
H5 R	CACTGTAGCACTACTTTTGC
H6 F	**CTGAGCCTTGTGATGACT**
H6 R	TGAGTGGAACTGTACATCAG
**Human Amplicon NGS primers (5'→ 3')**	
H5 F<br>H5 R	AGCCTGATACGAATCTTCCTGA<br>GCGTTTTTAAAGCGTGTTCTCG
hOT1 F<br>hOT1 R	GACTACCCCAGGGTCTAGCATT<br>TTGTTATCCATGCCTTGCAG
hOT2 F<br>hOT2 R	CAAGCCAGTTTCTAAGGGTCA<br>TACACTTACAGCAGGACCCACT
hOT3 F<br>hOT3 R	AAAAGGGTTCCATCATTCCC<br>TGCCCCAAAGTACAAACAAG
hOT4 F<br>hOT4 R	TGTGATAGTTGCTTTGAGCC<br>AACTTCTAACCTGGTACTCCTGG
hOT5 F<br>hOT5 R	CTTCAAAGTGAAGCTGATGC<br>ATCAAGGCTTTGGCTTGC

OT = off-target; F=forward; R=reverse,
Sanger seq primers bolded for *M6, **M4-5

NGS primers with Illumina Adapter overhangs:
F CTACACGACGCTCTTCCGATCT
R AGACGTGTGCTCTTCCGATCT

Supplementary Table 2. RT-PCR Primers

	Gene Name	Forward (5'→3')	Reverse (5'→3')
Mouse	*36B4*	TCCAGGCTTTGGGCATCA	CTTTATCAGCTGCACATCACTCAGA
	Nrip1	AGACCAGAACTTTAACCTCTCGG	CGATGGAATCAGACAGCCTCT
	Ucp1	ACTGCCACACCTCCAGTCATT	CTTTGCCTCACTCAGGATTGG
	Cidea	ATCACAACTGGCCTGGTTACG	TACTACCCGGTGTCCATTTCT
	Pgc1α	TATGGAGTGACATAGAGTGTGCT	CCACTTCAATCCACCCAGAAAG
	Cox7a	GCTCTGGTCCGGTCTTTTAGC	GTACTGGGAGGTCATTGTCGG
	Cox8b	TGTGGGGATCTCAGCCATAGT	AGTGGGCTAAGACCCATCCTG
	Glut4	CAATGGTTGGGAAGGAAAAGGGCTA	GTAGGCGCCAATGAGGAACCGTC
	Pepck1	ATGAAGTTTGATGCCCAAGGCAAC	GGATTTGTCTTCACTGAGGTGCC
	Fasn	GGAGGTGGTGATAGCCGGTAT	TGGGTAATCCATAGAGCCCAG
	Nrg4	CACGCTGCGAAGAGGTTTTTC	CGCGATGGTAAGAGTGAGGA
	Mcp1	TAAAAACCTGGATCGGAACCAA	GCATTAGCTTCAGATTTACGGGT
	Otop1	ACCTGCTGTGCGTTCTAACC	CGAACAACGTGATGCTTCCG
	Adck3	GCCCAGAGCATTAACAGTGAC	CTCAAGGGTAAGCTCCCGTC
	Slc25a42	CCCTGGACCGGACCAAGAT	TGTATTCTTCGTGTGCGCTGA
	Acaa2	CTGCTACGAGGTGTGTTCATC	AGCTCTGCATGACATTGCCC
	Letmd1	GCTCTGTGGGGTTCGACTG	GGTCACCACGTAAGAAATCAAGT
	Fabp3	ACCTGGAAGCTAGTGGACAG	TGATGGTAGTAGGCTTGGTCAT
	Pank1	GTTCGCCCAGCATGATTCTC	CTTAACCAGGGTTCCACCGAT
	Gpd1	ATGGCTGGCAAGAAAGTCTG	CGTGCTGAGTGTTGATGATCT
	Gpd2	GAAGGGGACTATTCTTGTGGGT	GGATGTCAAATTCGGGTGTGT
	Cpt1b	GACTTCCGGCTTAGTCGGG	GAATAAGGCGTTTCTTCCAGGA
	Gyk	TGAACCTGAGGATTTGTCAGC	CCATGTGGAGTAACGGATTTCG
	Pdk4	AGGGAGGTCGAGCTGTTCTC	GGAGTGTTCACTAAGCGGTCA
	Car4	TACGTGGCCCCCTCTACTG	GCTGATTCTCCTTACAGGCTCC
	Pat2/ Slc36a2	AAGCTCCTGTATGTCGTCGGT	CCGCAGGGACGTAGAACTG
	P2rx5	TGGAAGGGGTTCGTGTTGTC	AGGGAAGTGTCAATGTCCTGA
	Tnfa	CCCTCACACTCAGATCATCTTCT	GCTACGACGTGGGCTACAG
	Il1β	CCCTGCAGCTGGAGAGTGTGGA	TGTGCTCTGCTTGTGAGGTGCTG
	Cd36	ATGGGCTGTGATCGGAACTG	GTCTTCCCAATAAGCATGTCTCC
	Locus 1	CACAAAGAGGAAGACCAG	TACTGACAGTGGGACCAT
	Locus 2	AAGAGGCTGTCTGATTCC	AGCCTAGAGCTGAATGACT
	Locus 3	GCTTTGTTGAGTCATCCC	CAGGCAACACTAGGCTTT
	Locus 4	GGACTGATGTATAGTTCCCC	CTATGATCCGTCACACAAC
Human	*RPL4*	GCCTGCTGTATTCAAGGCTC	GGTTGGTGCAAACATTCGGC
	NRIP1	TAGCACATTACTGGCCTC	CTTGAGGCTCTGCCTGATTT
	UCP1	AGAAGGGCGGATGAAACTCT	ATCCTGGACCGTGTCGTA
