# Supplementary material for: CRISPR-enhanced human adipocyte browning as cell therapy for metabolic disease
Source: Nat Commun. 2021 Nov 26;12:6931. doi: 10.1038/s41467-021-27190-y (PMC8626495; doi:10.1038/s41467-021-27190-y)
Supplement: Supplementary file 3 — Reporting Summary [file 41467_2021_27190_MOESM3_ESM.pdf]

nature research

Corresponding author(s): Micheael P. Czech and Silvia Corvera

Last updated by author(s): Oct 21, 2021

Reporting Summary

Nature Research wishes to improve the reproducibility of the work that we publish. This form provides structure for consistency and transparency in reporting. For further information on Nature Research policies, see our Editorial Policies and the Editorial Policy Checklist.

Statistics

For all statistical analyses, confirm that the following items are present in the figure legend, table legend, main text, or Methods section.

n/a	Confirmed	
☐	☒	The exact sample size (*n*) for each experimental group/condition, given as a discrete number and unit of measurement
☐	☒	A statement on whether measurements were taken from distinct samples or whether the same sample was measured repeatedly
☐	☒	The statistical test(s) used AND whether they are one- or two-sided<br>*Only common tests should be described solely by name; describe more complex techniques in the Methods section.*
☐	☒	A description of all covariates tested
☐	☒	A description of any assumptions or corrections, such as tests of normality and adjustment for multiple comparisons
☐	☒	A full description of the statistical parameters including central tendency (e.g. means) or other basic estimates (e.g. regression coefficient) AND variation (e.g. standard deviation) or associated estimates of uncertainty (e.g. confidence intervals)
☐	☒	For null hypothesis testing, the test statistic (e.g. *F*, *t*, *r*) with confidence intervals, effect sizes, degrees of freedom and *P* value noted<br>*Give P values as exact values whenever suitable.*
☒	☐	For Bayesian analysis, information on the choice of priors and Markov chain Monte Carlo settings
☒	☐	For hierarchical and complex designs, identification of the appropriate level for tests and full reporting of outcomes
☒	☐	Estimates of effect sizes (e.g. Cohen's *d*, Pearson's *r*), indicating how they were calculated

Our web collection on statistics for biologists contains articles on many of the points above.

Software and code

Policy information about availability of computer code

Data collection	IGV_2.5.3, SnapGene Viewer 5.1.6
Data analysis	GUIDE-seq analysis pipeline Release 3.13, DolphinNext RSEM v.1.3.1, DESeq2 v.1.18.1, biomaRt v 2.44.2, CRISPResso2 v2.0.20b, Prism GraphPad 9, ImageJ v.1.51

For manuscripts utilizing custom algorithms or software that are central to the research but not yet described in published literature, software must be made available to editors and reviewers. We strongly encourage code deposition in a community repository (e.g. GitHub). See the Nature Research guidelines for submitting code & software for further information.

Data

Policy information about availability of data

All manuscripts must include a data availability statement. This statement should provide the following information, where applicable:

- Accession codes, unique identifiers, or web links for publicly available datasets
- A list of figures that have associated raw data
- A description of any restrictions on data availability

All sequencing data that support the findings of this study have been deposited in the NIH Sequence Read Archive via BioProject PRJNA745932. Other source data are provided with this paper as a Source Data file. Source data are provided with this paper.

Field-specific reporting

Please select the one below that is the best fit for your research. If you are not sure, read the appropriate sections before making your selection.

- [x] Life sciences
- [ ] Behavioural & social sciences
- [ ] Ecological, evolutionary & environmental sciences

For a reference copy of the document with all sections, see nature.com/documents/nr-reporting-summary-flat.pdf

Life sciences study design

All studies must disclose on these points even when the disclosure is negative.

Sample size	Sample sizes were determined at the start of each cohort for all in vivo work. We performed multiple experiments with varying cohort sizes to strengthen the reproducibility of the data. The experiments require many cells and starting material, therefore it was necessary to perform several experiments with smaller n, but following the exact same protocol. In both in vitro and in vivo samples, the data were highly reproducible. In order to calculate statistical significance in the in vitro, we secured a minimum of 3 biologically independent replicates. In the implantation studies, we conducted independent cohort each including all conditions (control and NRIP1KO) with a number of animals ranging 2-6 per condition. The fact that our data and observations were reproducible in the separate cohorts, gave us confidence that our sample size is adequate. Sample size calculations were not performed prior to starting the experiment, however in most in vivo experiments we have routinely used physiological measurements such as glucose tolerance tests, body weight measurements and effects on liver clearing of triglyceride. We have developed a method that allows us to measure the glucose in one cohort on the same day which limits the total number of mice used at one time, although in each cohort we have several of each condition. We have utilized the method of repeating experimental results in completely separate cohorts of mice during the period of an entire year or more to ensure our data is consistent and statistically significant. From previous experiments we know that glucose tolerance tests in our animals become statistically significant when 5 animals per group are used. We used a highier number than that as mice were split post mortem for different analysis.
Data exclusions	No data were excluded.
Replication	All experiments have been repeated under biologically independent conditions by more than one researcher in many cases. The number and kind (biological or technical) replicates have now been clarified in the manuscript. All in vitro data presented reflect independently executed experiments in biologically independent cell lines deriving from different mice with reproducible results. The in vivo data represent biologically independent mice with showing a very reproducible phenotype. The cohorts were repeated three times and each cohort included both conditions presented with consistent data and statistically significant.
Randomization	In the implantation studies, the recipients were randomized. Research randomizer online tools were used for that purpose. In the in vitro studies, we describe primary adipocytes that were transfected in different conditions. Randomization does not apply in this process.
Blinding	Blinding was used for the analysis of the GUIDE-seq results. The investigator who performed the analysis (TR) was different to the investigator who prepared the library (ET) in GUIDE-seq and Amplicon NGS. The analysis investigator was blinded to the experimental conditions corresponding to the sequencing files.

Reporting for specific materials, systems and methods

We require information from authors about some types of materials, experimental systems and methods used in many studies. Here, indicate whether each material, system or method listed is relevant to your study. If you are not sure if a list item applies to your research, read the appropriate section before selecting a response.

Materials & experimental systems

n/a	Involved in the study	
[ ]	[x]	Antibodies
[ ]	[x]	Eukaryotic cell lines
[x]	[ ]	Palaeontology and archaeology
[ ]	[x]	Animals and other organisms
[x]	[ ]	Human research participants
[x]	[ ]	Clinical data
[x]	[ ]	Dual use research of concern

Methods

n/a	Involved in the study	
[x]	[ ]	ChIP-seq
[x]	[ ]	Flow cytometry
[x]	[ ]	MRI-based neuroimaging

Antibodies

Antibodies used	Antibodies used in the study include: UCP1-Abcam#10983, Rabbit polyclonal, LOT GR3215381-15 1:700;<br>Rip140-Millipore #MABS1917, Mouse Monoclonal [2656C6a], LOT 3397921,1:1000,<br>Tubulin-Sigma #T5168, Mouse monoclonal [B-5-1-2], LOT 039M4769V, 1:4000;<br>GAPDH-Cell Signaling #21185, Mouse monoclonal [6C5], LOT 14, 1:1000;<br>SpyCas9-Cell Signaling #19526S, LOT 1, 1:5000,<br>OXPHOS Abcam #110413, LOT GR3336290-11,1:1000 which includes the following Mouse monoclonals: [20E9DH10C12] to NDUFB8,

	[21A11AE7] to SDHB, [13G12AF12BB11] to UQCRC2,[1D6E1A8] to MTCO1, [15H4C4] to ATP5A - Mitochondrial Marker and as positive control is contains Rat heart tissue lysate - mitochondrial extract (contains SDS/DTT) (ab110341),<br>Rabbit IgG Millipore #12-370, LOT 3456023
Validation	UCP1 Abcam#10983 antibody was validated by including brown adipose tissue on all blots because UCP1 is highly expressed in brown adipose tissue. RIP140 Millipore #MABS1917 antibody was validated by detecting the knock-down of protein in the NRIP1-CRISPR samples. RIP140 antibody was further validated by the use of a Rip140-HA over-expressing construct to confirm molecular weight of band detected. Rabbit non-immune IgG Millipore #12-370 is used as a negative control for the RIP140 protein immunoprecipitation samples. The concentration of Rabbit non-immune IgG was used at the same concentration as Rabbit anti-Rip140. Both antibodies were used at a concentration we have listed in our laboratory immunoprecipitation protocols. Tubulin Sigma #T5168 and GAPDH Cell Signaling #21185 were both used as protein loading controls and have been reported many times in the literature. Loading control antibodies were used at dilutions suggested by the manufacturer. SpyCas9 Signaling #19526S antibody was used and detected SpyCas9 in samples with purified SpyCas9 protein, vs 48hr+ where it is degraded. The disappearance of SpyCas9 was validation that the antibody was recognizing the appropriate protein. Oxphos Abcam #110413 antibody also used with a brown adipose tissue sample as a positive control due to the high mitochondria and oxidative phosphorylation proteins in brown adipose tissue.

Eukaryotic cell lines

Policy information about cell lines

Cell line source(s)	Murine ells used in this manuscript were primary mouse preadipocytes isolated from the stromal vascular fraction of the inguinal adipose tissue of 3-4 week old mice. Human cells in this manuscript were cell lines of human adipocyte progenitors established at the Corvera Lab and provided to our group de-characterized.
Authentication	Authentification of the cell lines included running RT-PCR on samples to look for expected gene profiles of mature adipocytes or fibroblasts.
Mycoplasma contamination	Cell lines were not tested for mycoplasma.
Commonly misidentified lines<br>(See ICLAC register)	No such cell line was used.

Animals and other organisms

Policy information about studies involving animals; ARRIVE guidelines recommended for reporting animal research

Laboratory animals	All animal work was approved by the University of Massachusetts Medical School Institutional Animal Care Use Committee (IACUC protocol no.1600 to Michael P. Czech and no. 2007 to Silvia Corvera) with adherence to the laws of the United States and regulations of the Department of Agriculture. Mice were housed at 20-22 °C on a 12-hour light/12-hour dark cycle with ad libitum access to food and water. C57BL/6J male mice were purchased from Jackson Laboratory for implant studies. C57BL/6J (Jackson Laboratory) male mice were bred for primary preadipocyte cultures. Briefly, 10-week old male mice arrived and were allowed to acclimate for a week prior to any procedures.<br>Male NOD.Cg-Prkdcscid Il2rgtm1Wjl/SzJ (denoted as NSG) mice were kindly donated by Taconic Biosciences, Inc. At 11 weeks of age NSG mice received implants with edited primary human adipocytes. Mice were maintained on a chow diet for the first 10 weeks, followed by placing them at thermoneutral environment with a 60 kcal% high fat diet (Research Diets, D12492i) for the remainder of the experiment from 10 to 15 weeks post implant. Housing under thermoneutrality was achieved by placing the NSG mice at 30°C on a 12-hour light/12-hour dark cycle.
Wild animals	No wild animals were used.
Field-collected samples	No field collected samples were used.
Ethics oversight	University Massachusetts Medical School Institutional Animal Care and Use Committee approved all experiments involving animals in this work.

Note that full information on the approval of the study protocol must also be provided in the manuscript.
